# A Battle Lost? Report on Two Centuries of Invasion and Management of *Lantana camara* L. in Australia, India and South Africa

**DOI:** 10.1371/journal.pone.0032407

**Published:** 2012-03-05

**Authors:** Shonil A. Bhagwat, Elinor Breman, Tarsh Thekaekara, Thomas F. Thornton, Katherine J. Willis

**Affiliations:** 1 School of Geography and the Environment, University of Oxford, Oxford, United Kingdom; 2 Biodiversity Institute, Oxford Martin School, Department of Zoology, University of Oxford, Oxford, United Kingdom; 3 Environmental Change Institute, School of Geography and the Environment, University of Oxford, Oxford, United Kingdom; 4 Department of Biology, University of Bergen, Bergen, Norway; Institut Mediterrani d'Estudis Avançats (CSIC/UIB), Spain

## Abstract

Recent discussion on invasive species has invigorated the debate on strategies to manage these species. *Lantana camara* L., a shrub native to the American tropics, has become one of the worst weeds in recorded history. In Australia, India and South Africa, Lantana has become very widespread occupying millions of hectares of land. Here, we examine historical records to reconstruct invasion and management of Lantana over two centuries and ask: Can we fight the spread of invasive species or do we need to develop strategies for their adaptive management? We carried out extensive research of historical records constituting over 75% of records on invasion and management of this species in the three countries. The records indicate that governments in Australia, India and South Africa have taken aggressive measures to eradicate Lantana over the last two centuries, but these efforts have been largely unsuccessful. We found that despite control measures, the invasion trajectory of Lantana has continued upwards and that post-war land-use change might have been a possible trigger for this spread. A large majority of studies on invasive species address timescales of less than one year; and even fewer address timescales of >10 years. An understanding of species invasions over long time-scales is of paramount importance. While archival records may give only a partial picture of the spread and management of invasive species, in the absence of any other long-term dataset on the ecology of Lantana, our study provides an important insight into its invasion, spread and management over two centuries and across three continents. While the established paradigm is to expend available resources on attempting to eradicate invasive species, our findings suggest that in the future, conservationists will need to develop strategies for their adaptive management rather than fighting a losing battle.

## Introduction

Biologists, ecologists and conservationists disagree on the best way to respond to invasive species [1]–[5]. *Lantana camara* L. (referred to as Lantana from here on), a shrub native to the American tropics, has become one of the worst weeds in the world. Lantana was introduced in early-mid 19^th^ century in tropical parts of Africa, Asia and Oceania as an ornamental garden plant [6]–[8]. Global Invasive Species Information Network now identifies Lantana among the top ten invasive species in the world based on the number of countries where these species are considered invasive [9] and IUCN's list of world's 100 worst alien invasive species includes Lantana [10]. In Australia, India and South Africa, Lantana has been reported as a widespread weed [11], [12]. Management of invasive species is highlighted as a major task facing conservation planners [13], [14] and accordingly governments and some non-governmental actors in these countries are continuing to take aggressive measures to attempt to eradicate Lantana. However, the knowledge of long-term trends of invasive species is very limited due to the lack of historical records. The majority of studies on invasive species address timescales of less than one year; and fewer than 10% address timescales of >10 years [15]. Strayer et al. [15] examined *c.* 200 studies published between 2001 and 2005 in ecological journals and concluded that “most studies of the effects of invasive species have been brief and lack a temporal context” (p. 645). An understanding of species invasions over long time-scales is therefore of paramount importance [16]. Here, we examine the invasion and adaptive management of Lantana over two centuries using historical records. The majority of these records were held in the Bodleian Libraries at Oxford, and a few in government libraries in South India. These consisted primarily of regional and national forestry reports and journals, together with selected botanical records, conference reports and government publications.

### Spread of Lantana and its current status

Current estimates suggest that Lantana has invaded more than 5 million ha in Australia, 13 million ha in India and 2 million ha in South Africa [17]–[20]. Reports of this invasive from the 19^th^ and 20^th^ centuries from these three countries give an insight into the spread of Lantana [21], [22]. Its introduction to botanical gardens in European colonies made it a popular garden and hedge plant in the early 19^th^ century [23]–[25]. Soon afterwards, e.g. in Australia in the 1850s, Lantana was even considered ‘naturalised’ in some reports [26]. In others, it was mentioned as merely present, e.g. [27], but was not perceived as a problem. It was only in the late 19^th^ century in Australia [28] and India [29], and mid-20^th^ century in South Africa (reported in [30]), that Lantana was considered as an invasive or noxious weed. Active management of Lantana began in the early 20^th^ century in Australia, e.g. [31], and India, e.g. [32], and in the late 20^th^ century in South Africa, e.g. [33]. Various methods of controlling Lantana were trialled throughout the mid-20^th^ century, including control with fire, mechanical removal, chemical and biological control; and reports suggest that these methods or their combination was successful in some regions, e.g. [33]–[35]. However, reports from the latter part of the 20^th^ century suggest that Lantana continued to spread despite management [36]–[38].

### Efforts to eradicate Lantana

Historical records indicate that the drive to eradicate Lantana demanded substantial resources and manpower throughout the 19^th^ and 20^th^ centuries. A combination of fire, mechanical and biological control was used in India as early as 1921 [32]. In Australia control of Lantana is reported in the early part of the 20^th^ century and in South Africa in the late-20^th^ century, but the number of reports about control increase in the 1970s in Australia [39] and in South Africa [40] suggesting that substantial effort was made to eradicate Lantana around this time. Reports from Australia suggest that emphasis was on biocontrol – to reduce Lantana to a level below a threshold of impact. For example, the Forestry Commission of New South Wales reports from 1959 through to 1983 provide details on insects used in biocontrol and their impact [41]. In South Africa, on the other hand, the emphasis was on mechanical removal. For example, the Department of Forestry [42] reports the heavy cost of removal in 1981 and a wide variety of methods used including mowing, hoeing out, drying and burning. Despite substantial weed management efforts, Lantana still remains a major concern in Australia, India and South Africa [22].

Here we reconstruct invasion trajectories of Lantana in Australia, India and South Africa based on historical records from the 19th and 20th centuries. We ask: (a) What were the drivers of spread of Lantana? (b) How rapidly did Lantana spread and why? (c) What attempts were made to control the spread of Lantana and were they successful? In answering these questions, and in the absence of a review protocol to study invasive species from historical records, we develop a novel method to reconstruct invasion trajectories.

## Results

**Figure 1:**
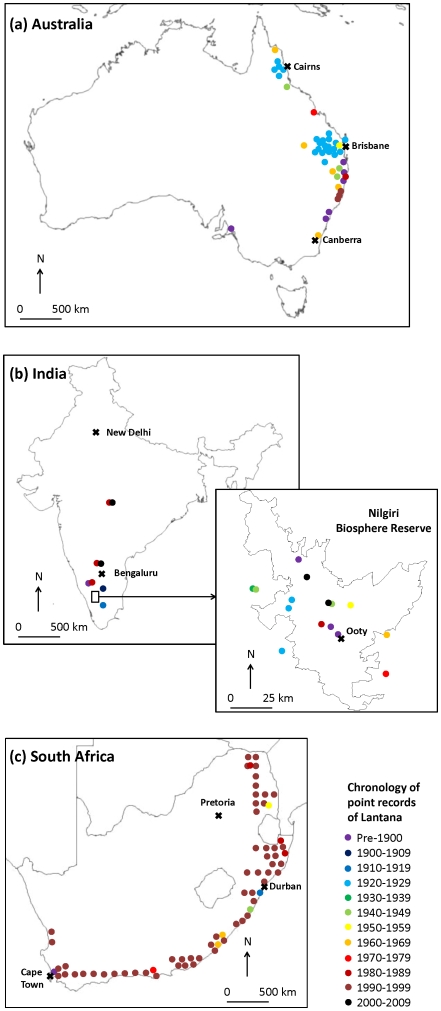
Spatial distribution of Lantana records from Australia, India and South Africa based on historical reports of its spread, management and control. A total of 42 points records are mapped from (**a**) Australia, 23 from (**b**) India and 10 from (**c**) South Africa. Records from South Africa between 1990–1999 come from one source [97]. Early records suggest spread of Lantana around towns and cities where it was first introduced. Later records indicate its spread in the wider countryside despite management. Most Indian records come from Nilgiri Biosphere Reserve where a more regional analysis of Lantana invasion was carried out.

The spatial maps of point data on Lantana spread suggest that the early invasion is recorded around towns and cities; and it subsequently spreads into the wider countryside (Fig. 1A, 1B, 1C). For example, records of invasion in Australia suggest that in the 1920s Lantana spread in areas around Brisbane and Cairns, and subsequently in the countryside along the Queensland coast. In South Africa, initial records of invasion are from Durban and Cape Town, while later records suggest the spread of Lantana along the eastern and southern coast. Our Indian data are mainly focused around the Nilgiri Biosphere Reserve in South India and are at a much finer regional scale. However, even at this scale, the records suggest initial spread of Lantana around cities such as Ooty, followed by its invasion in to the wider countryside, although this spatial pattern is not as clear-cut as in Australia or in South Africa.

**Figure 2:**
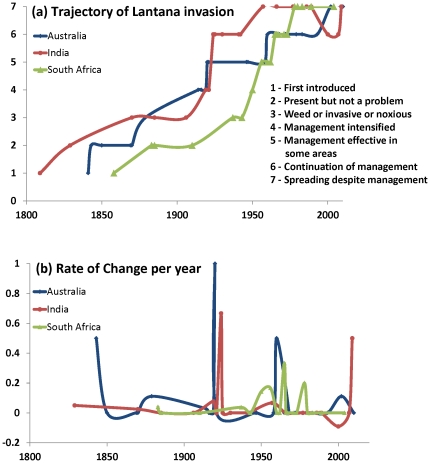
Invasion trajectories of Lantana in Australia, India and South Africa. (**a**) Historical records of Lantana spread, control and management are scored on a scale of 1–7: (1) first introduced; (2) present, but not a problem; (3) considered weed, invasive or noxious plant; (4) management intensified; (5) management reported effective in some areas; (6) continuation of same management strategy; (7) Lantana seen to be spreading in spite of management. (**b**) Rate of change is calculated as increase per year in the state of invasion, measured on the seven-point scale.

A quantitative assessment of the scale of invasion of Lantana gives an insight into its invasion trajectory between 1800 and the present day. The overall trend of Lantana invasion in Australia, India and South Africa is similar showing a consistent increase throughout the time period in question (Fig. 2A). There are, however, some regional differences. The time of introduction is different across the countries, with Lantana being introduced to India shortly after 1800, to Australia shortly before 1850 and to South Africa shortly after 1850. Lantana is soon considered a ‘weed’ in historical records, but the need for its management appears to arise in India and Australia only in the 1920s, while in South Africa, management is not introduced until after the 1950s. The management of Lantana continues thereafter, but recent reports from each country indicate that this plant is spreading despite management efforts to eradicate or control it. There is a small reversal in the trend in India around 2000s, reflected by the fact that management maintains status quo and, significantly, managers no longer mention eradication of Lantana as a goal but rather only mitigative management for control of the plant such that it does not adversely affect wildlife. However, subsequent reports suggest that Lantana is continuing to spread despite such management.

Lantana invasion trajectory shows its highest rate of change in the 1920s in Australia and India (Fig. 2B). In South Africa, the highest rate of change is around the 1960s which coincides with another surge in reports of the species in Australia around the same time. The rate of change in India falls momentarily just before 2000s, probably reflecting the shift in focus from management for eradication to management for control, but increases subsequently.

**Figure 3:**
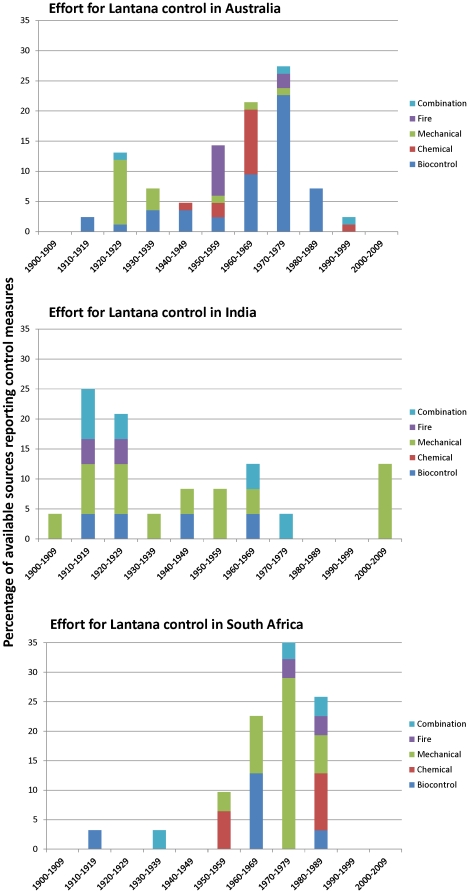
Methods used for control, management and eradication of Lantana during the 20^th^ century. A variety of methods are reported in historical records: fire, mechanical removal, chemical and biological control and a combination of these four measures. A total of 84 reports on control measures are available from Australia, 31 from South Africa and 24 from India. For parity in comparison across the three countries, frequencies of reports are expressed as percentages.

Control measures of Lantana include fire, mechanical removal, chemical and biological control or their combination (Fig. 3). While the peak in control effort in India is seen in the 1910s, in Australia and South Africa, the peak is seen in the 1970s. Biocontrol appears to be the most prominent method in Australia during this time; and in South Africa reports suggest mechanical removal as preferred method. In India, a combination of methods except chemical control is used with a majority of reports indicating mechanical removal – including the use of domestic elephants to uproot Lantana – as the preferred method.

**Figure 4:**
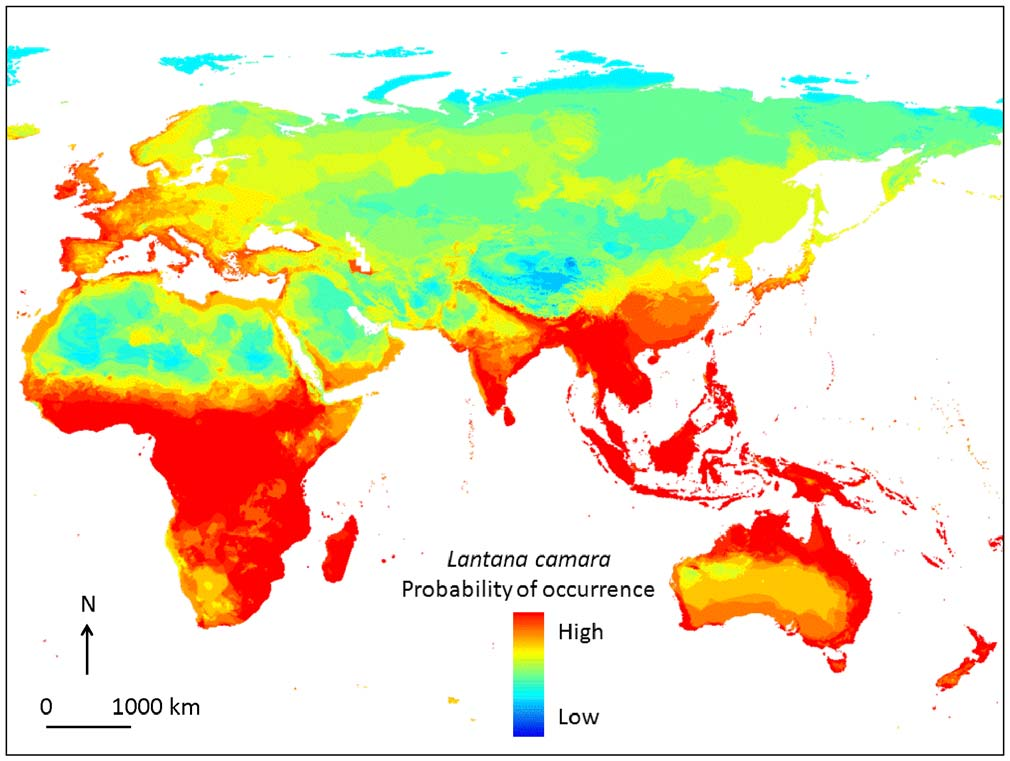
Bioclimatic niche model of Lantana camara based on point data stored in the Global Biodiversity Information Facility and generated by automated openModeller algorithm. The point data are derived by searching the Global Biodiversity Information Facility [90] for *Lantana camara*, which has the following recognised synonyms: *Lantana aculeata*, *Lantana tiliifolia*, *Lantana camara* var. nivea, *Lantana camara* var. mista, *Lantana camara* var. mutabilis, *Lantana camara* var. hybrida, *Lantana camara* var. flava, *Lantana camara* var. aculeata, *Lantana camara* var. sanguinea. The openModeller niche model uses WorldClim global climate layers (climate grids) with a spatial resolution of one square kilometre [91].

A bioclimatic niche model of Lantana (Fig. 4) indicates that a substantially greater area than Lantana's current distribution falls within its bioclimatic envelope. This includes most of the sub-Saharan Africa, most of peninsular India and large tracts along the northern and eastern coast of Australia, as well as South East Asia. In addition, parts of the Mediterranean basin and coastal parts of Western Europe also fall within Lantana's bioclimatic envelop.

## Discussion

### The effect of Lantana on ecosystems

While native to the Americas, Lantana was brought to Europe in the 16th century and since that date it has been subjected to horticultural improvement through selection of traits and hybridisation, leading to the creation of 630 named cultivars and variants. This genetically diverse artificial species complex was the source of introductions to India, Australia and South Africa [6]. This plasticity has enabled it to adapt to a wide variety of habitats – from sea level to 1800 m or more [43], [44]. Lantana grows in tropical, subtropical and temperate climates, with mean annual rainfall of <1000–>4000 mm [45]. Lantana can also aggressively compete for surface-soil nutrients and water [46], is allelopathic and hinders seedling recruitment and growth of other plants in its vicinity [47]–[50], it produces abundant seed that is dispersed large distances by birds and water, and is able to form dense stands under favourable conditions, enabling it to quickly dominate native vegetation [8], [51], [52]. These properties have made Lantana one of the most successful weeds, which can dramatically transform ecosystems. Studies suggest that Lantana invasion affects local biodiversity and all four categories of ecosystem services – provisioning, regulating, supporting and cultural [53]. For example, Lantana is known to pose serious threat to biodiversity in several World Heritage sites and Endangered Ecological Communities in Australia (e.g. rainforest of northern Queensland, Fraser Island and the Greater Blue Mountains), the Fynbos of South Africa, and biodiversity hotspots in India (e.g. the Western Ghats and Eastern Himalayas) [12], [54], [55]. Furthermore, Lantana is toxic to livestock and harbours the tsetse fly, the vector of African sleeping sickness, and malarial mosquito [12]. It is also known to affect economic viability of 14 major crops around the world including coffee, tea, rice, cotton, oil palm, coconut and sugarcane, in part due to its allelopathic properties, which reduce productivity of crop plants [22].

### Invasion trajectory and management effort

Our results suggest that Lantana has continued an upward trajectory of spread and invasion in Australia, India and South Africa. One striking feature of this invasion trajectory is its spread despite intensive management. For example, in the early 1980s Lantana had invaded about 2.2 million hectares of forest plantations, watercourses and savannah in South Africa and mechanical and chemical control had little effect on this invasive species [43]. Similarly, in India all efforts using biological control in the mid-1980s had failed and Lantana was still spreading [38]. In an assessment of invasive species in Queensland, Australia in 2003 Lantana was ranked the most invasive weed [56]. It is also evident that considerable resources were spent on Lantana control and management in all three countries while the invasion trajectory continued to rise upwards. In 1973, for example, the cost of Lantana control in Queensland, Australia was estimated at *c.*A $1 M (US $1 M) per year [57]. In South Africa, the cost of chemical poisoning to control Lantana was estimated at R 1.7 M (US $ 250,000) per year in 1999 [58]. Estimates from India suggest that the present cost of Lantana control is approximately INR 9000 (US $ 200) per hectare [59]. In addition, substantial opportunity costs of Lantana invasion are reported in the literature; for example, Lantana's global infestation of millions of hectares of grazing land [22], [60]. A study of the grazing sector in Queensland, Australia suggested that in 2007 this sector incurred opportunity costs of A $ 121 M (US $ 121 M) due to Lantana invasion [61] in comparison with A $ 3 M (US $ 3 M) per year loss recorded for the same sector in 1985 [60]. Similar concerns have also been reported from India [22].

### Rate of change and drivers of spread

Our invasion trajectories of Lantana across three continents suggest that there were episodes of rapid change. In Australia, Lantana was first introduced to the old Botanical Gardens in Adelaide, South Australia, and due to its popularity as garden plant multiple introductions followed, mainly in New South Wales and Queensland [62], [63]. In India, Lantana is known to have been introduced in 1807 in Kolkata botanical gardens [64] while in the Nilgiris, where we focused our investigation, Lantana was mentioned for the first time by Hough in 1829 [24]. In South Africa, the first introduction did not take place until 1858, when it was introduced to Cape Town [25], [65]. The invasion trajectory of Lantana in Australia and India shows a rapid rate of change around mid-1920s. This was shortly after the end of World War I, when a period of post-war economic depression occurred [66]. Both Australia and India experienced rapid land use change, mining and exploitation of other natural resources at this time [67]. This might have triggered the spread of Lantana on both continents. Paradoxically, reports of Lantana control in India from the 1910s and 1920s suggest that this is also the time when the greatest effort to control Lantana was made and a variety of control measures were used (Fig. 3). South Africa did not experience such a rapid rate of change at that time possibly because Africa was still relatively isolated from extractive resource industries and large-scale intensive agriculture, e.g. [68]. South Africa, however, experienced three episodes of rapid rate of change following World War II (between 1950 and 1970). Australia also experienced a similar increase in the 1960s. Both these increases might be related to post-war land use change in these countries [69], [70]. Such a rise is not apparent in India, possibly because the land tenure and land use in the Nilgiris, where our data comes from, has not witnessed any major changes since the 1950s [71], [72]. Reports on Lantana control suggest that it was in the 1970s in Australia and South Africa that the greatest effort to control Lantana was invested (Fig. 3). While the South African trajectory continued to rise at a steady rate of change between 1970s and 2000s (possibly evidence that some of the control measures were working), Australia and India experienced another surge in 2000s (Fig. 2B). This might be a consequence of recent land use pressure on expanding agricultural sectors in both countries and consequent land use change [73], [74]. These episodes of increase in the rate of change indicate the change in land use as possible driver of Lantana spread [75], [76]. Lantana is a shade intolerant plant [8] and therefore any increase in the intensity of land management, e.g. increase in farmland area or opening up of forests, would have facilitated its spread. Similarly, any lapse in land management would have led to an increase in marginal lands where Lantana could have invaded as it is known to colonise rapidly after fire or to invade cleared grazing areas and forest plantations [7], [77]–[79].

### Role of Lantana in providing ecosystem function and livelihoods

The rapid spread of Lantana is evident from its invasion trajectory, but does this mean that its spread has always had detrimental effects on the ecosystems and the local communities who depend on them? Lantana has several negative impacts on ecosystems, but its positive role has also been documented. For example, while Lantana is known to compete with forestry species and reduce their productivity [52], it can also increase the regeneration of some non-timber forest products [12]. In addition while the presence of Lantana, a bee-pollinated plant [80], reduces pollinator load of native plants [81], it makes a useful honey plant [82]. Lantana's toxic effects on livestock and its allelopathic effects on other plants are also well documented [47]–[50], however, its alkaloids are also known to have anti-bacterial, anti-microbial, anti-inflammatory, anti-tumour, and anti-AIDS properties that have the potential for use in medicine [83]. In comparison with grass-covered surfaces, Lantana cover can increase water run-off and, therefore, surface soil erosion, but it has also proven useful to prevent soil erosion on barren mountain slopes and in deforested areas [84], [85]. Interestingly, in India, many forest managers now accept Lantana as a naturalised plant that plays an important role in the functioning of ecosystems by, for example, providing cover to carnivores, food for birds as well as some wild herbivores in addition to the livelihood benefits that Lantana provides to the local communities [86]. As such, they only aim to manage or control Lantana rather than attempting to eradicate it. Thus the change in management strategy from eradication to control and acceptance of Lantana reflects not only a realisation of the futility of eliminating Lantana altogether, but also increasing cognisance of its ecosystem effects, both positive and negative.

### From eradication to adaptive management

The focus of Lantana management thus far has been on its control and eradication. As indicated by the increase in the number of reports on Lantana control in the 1970s, substantial effort was made to control and eradicate Lantana in Australia and South Africa around this time [39], [40]. While the emphasis in Australia was on biocontrol [41], in South Africa mechanical removal was a preferred option [42]. Although these reports indicate substantial weed management efforts, they seem to have had little effect on the spread of Lantana [38], [43] and it still remains a major concern in Australia, India and South Africa [22]. The rapid invasion of Lantana has even instigated legislation for its control in Australia and South Africa [26], [87]. This legislation restricts its import and outlines rules for its eradication. In Australia, for example, Lantana is a declared Noxious Weed under the New South Wales Noxious Weeds Act 1993. All Lantana species are declared Class 3 plants under the Land Protection (Pest and Stock Route Management) Act 2002. Lantana species cannot be sold or distributed and landholders may be required to control these plants if they pose a threat to an environmentally significant area in Australia [88]. Similarly in South Africa, Lantana is a proclaimed noxious weed under the Weeds Act (No 42, 1937), and the owner or occupier of the property is obliged to eradicate Lantana when such a notice has been served [30]. The Conservation of Agricultural Resources Act (1983) in South Africa has subsequently declared Lantana as Category 1 invasive species, which must be eradicated or effectively controlled on farm units (The Conservation of Agricultural Resources Act – Act No 43, 1983). In comparison to Australia and South Africa no such legislation exists in India, but evidence suggests that instead local communities have adapted to the presence of Lantana. For example, a whole new cottage industry has sprung up in areas where Lantana is now abundant. This includes its use in basketry; making rubbish bins, flower pots and fruit plates; thatching roofs; weaving hedges and making toys and furniture [22], [89]. On a more industrial scale, Lantana pulp is used for making paper in India [90]. Adaptive management is an iterative, ongoing process of learning and responding to environmental conditions while acknowledging their dynamics, uncertainty, and changes over time [91]. The adaptations to Lantana in India represent both autonomous and planned attempts by human groups to innovate and diversify their livelihoods in response to the increasing abundance of Lantana. Further investigations are currently underway in the Western Ghats to see what other adaptation pathways, including practical measures of control, are being pursued by various groups in response to Lantana.

It is apparent that Lantana is an invasive plant that has adapted very well to the ecosystems it has invaded, often transforming their natural state. Furthermore, its bioclimatic niche and therefore potential for its expansion might include much more land area in Australia, India and South Africa than it has currently invaded (Fig. 4). While legislation and management have aimed at controlling the density and spread of Lantana, there is limited evidence for success of such control measures. The focus of legislation and management so far has been on Lantana's ‘ecosystem dis-services’, but there is also evidence that it provides certain ecosystem services and livelihoods. Furthermore, much of the recent scientific evidence suggests that invasive species are here to stay [1], [15], [16], [92]. For example, a long-term data set of naturalized plant species on islands [92] demonstrates that the mean ratio of naturalized to native plant species across islands has changed steadily for nearly two centuries, indicating that these new species assemblages have created novel ecosystems. In the future, conservationists and managers will need to grapple with the novel ecosystems that invasive species (such as Lantana) give rise to. In some areas, however, there will always be the need to control Lantana as it is a competitive weed, but these control measures need to be well defined and realistic. Given that the success of the eradication and management of Lantana has been limited thus far, better tools are needed to manage Lantana, possibly including more effective biological control agents. However, where such control measures are not practical, one way forward might be to embrace this pan-global invasive species and to find ways for its adaptive management.

### Conclusion

Calls have been made recently for conservationists to focus on the functional role of species in ecosystems rather than their origins: “Nearly two centuries on from the introduction of the concept of nativeness, it is time for conservationists to focus much more on the functions of species, and much less on where they originated” [1], p.154. We show that in Australia, India and South Africa, despite measures to control Lantana, its spread and invasion have continued. We do this by developing a quantitative scale for comparison of invasion trajectories across three continents. These invasion trajectories display rapid rates of change in the 1920s, between the two World Wars, possibly due to large-scale land use changes. Even though efforts to control Lantana peak in India in the 1910s and in Australia and South Africa in the 1970s, this has little effect on its invasion. For most invasive species, quantitative data on historical drivers of spread are lacking and therefore development of such quantitative scale can provide a better handle on drivers of their spread. Our long-term view of Lantana invasion across three continents suggests that the future management of invasive species will require an adaptive management approach to their invasion. Policymakers will need to find innovative and diverse approaches to such adaptive management whilst being prepared to embrace the novel ecosystems that invasive species create and to respond to future changes in social-ecological conditions that may evolve as a result of their presence. Such an adaptive management response will be most effective to improve the resilience of both ecosystems and societies to the presence of invasive species. In the future, therefore, managers will be much better off finding new ways to adapt to invasive species rather than fighting a losing battle to eradicate them.

## Materials and Methods

This investigation of Lantana's invasion trajectory is based on extensive research of historical records. We followed Preferred Reporting Items for Systematic Reviews and Meta-Analyses (PRISMA) guidelines [93] to identify, screen, determine eligibility and include reports in this analysis. We systematically surveyed between October 2010 and June 2011 reports in the Bodleian Libraries in Oxford published by forestry and land management departments in Australia, India and South Africa from the 1800s until the present day. Information sources included all forestry bulletins held for each country in the Oxford libraries, all microfiche forestry resources held for each country and any other relevant forestry documents that came to light in searching through this material. For India, forestry department reports and forest working plans held at government libraries in Bengaluru, Chennai and Nilambur were also searched in addition to all issues of Indian Forester held at Oxford libraries.

The eligibility criteria were deliberately broad in order to ensure that we included all relevant material. For Australia and South Africa, we searched reports from all regions where Lantana is reported. For India, we focused on the Nilgiris region in order to understand a regional-scale perspective of Lantana invasion and management. All reports that mentioned weeds and their control and mentioned Lantana in any capacity (for example taxonomic, occurrence, control measures, status) were included in the selection process. Similarly, report on a site situated within the study area in the study countries (for example, New South Wales and Queensland in Australia; Cape to Transvaal in South Africa; and Nilgiris in India) were also included. A thorough search of these records was carried out to examine the narrative surrounding Lantana in each record. Notes were made using direct quotes and paraphrasing; and any relevant citations given in reports were followed up for the verification of content.

Approximately 3000 records were thus identified through database searching (n = Australia 322; India 117; South Africa 125) and other sources (n = Australia 1350; India 173; South Africa 850). After duplicates were removed we were left with 1672 records for Australia, 290 for India and 975 for South Africa. All these records were screened for specific mention of Lantana and those that did not have a specific reference to Lantana were excluded (n = Australia 258; India 105; South Africa 100). We accessed approximately 2500 full-text articles for eligibility (n = Australia 1414; India 185; South Africa 875), again excluding those without specific reference to Lantana's management (n = 26 articles for Australia, 74 for India and 45 for South Africa). We used over 2000 reports (n = Australia 1388; India 111; South Africa 830) in the qualitative synthesis. We estimate that all the reports we included in this qualitative synthesis constitute at least 75% of historical literature on the introduction, spread and management of Lantana in these countries.

Out of the 116 reports shortlisted for quantitative analysis, a total of 53 were from Australia, 22 from India and 41 from South Africa. These reports presented spatial and temporal dimension of Lantana introduction, invasion, spread and control and were included in the quantitative analysis of the invasion trajectory (Table S1A, S1B, S1C). While the historical records may present only a partial picture of Lantana invasion and management, in the absence of any other ecological information going back to 1800s, the historical records we used provide an important insight into the invasion, spread and management of Lantana over two centuries and across three continents. An additional bias is likely to be introduced because the records come from forestry departments, who are interested in eradication of invasive species due to their economic impacts. The perception of forestry departments about the threat from invasive species therefore portrays only a partial picture of lantana invasion. However, in the absence of long-term historical ecological studies to verify such qualitative reports, we considered our semi-quantitative approach to be a pragmatic solution for an enhanced understanding of invasive species and their management.

To examine regions in Australia, India and South Africa that are affected by Lantana invasion, we plotted geographical co-ordinates for locations of all available reports (Fig. 1A, 1B, 1C). Where only place names were available, we derived geographical co-ordinates from Google Maps. We colour-coded these point data for each decade between 1900 and the present day. Very few records were available for the time period between 1800 and 1900, so we grouped these records into ‘pre-1900’ category.

The narratives of Lantana invasion included its mention as an ornamental plant, popular hedge plant, its spread as invasive; and the managers' success or failure to control it. A scale of 1–7 was used to score records along increasing severity of Lantana invasion (Fig. 2A): (1) first introduced; (2) present, but not a problem; (3) considered weed, invasive or noxious plant; (4) management intensified; (5) management reported effective in some areas; (6) continuation of same management strategy; (7) Lantana seen to be spreading in spite of management. The qualitative narratives were scored independently by two of the authors (EB and TT) to ensure that there is consistency in scoring. When scoring, the earliest record for each category was used to determine the timing to move up the scale. As such, ‘first introduced’ reflects the introduction of Lantana as ornamental plant and ‘present but not a problem’ reflects later narratives which do not yet refer to the plant as an invasive or weed, but report the presence of Lantana in the wider landscape beyond the areas where it was planted. Further up the scale, Lantana is considered a weed, invasive or noxious plant and is referred to as a problem. Management intensification reflects concerted effort by government authorities to control and manage the weed. Further up the scale, management is reported effective in some areas reflecting reports of management success. The continuation of the same management strategy is categorised as a separate entity because government agencies report using the same management strategy in the broader landscape, as opposed to intensifying management further. The final category, which reflects the spread of Lantana despite management captures reports of frustration from the government authorities that this weed is beyond control or management. The quantitative scale we devised allowed synthesis of anecdotal information reported in historical records and comparison of Lantana narratives across the three countries. The two authors (EB and TT) who scored each record independently arrived at the same score in 95% of the cases, indicating that our seven categories of the scale of invasion are robust. Where judgements differed, the two authors conferred their score before including it in the quantitative scale.

Based on this quantitative scale of Lantana invasion, we calculated rate of change per year (Fig. 2B) such that a sharp increase in the scale of invasion over time indicated high rate of change and slow increase in the scale of invasion indicated a low rate of change. A comparison of the rates of change allowed identification of time periods that coincided with rapid spread of Lantana.

To compare effort spent on controlling Lantana across the three countries, we examined the variety of methods reported for Lantana management. These methods were categorised into five classes: fire, mechanical, chemical, biocontrol and combination of all methods. We calculated percentage of total reports in each decade that mentioned each of these methods (Fig. 3).

To identify a potential bioclimatic envelop for Lantana, we used point data for Lantana camara (and synonyms) in Global Biodiversity Information Facility [94] and developed a global niche model for Lantana with automated openModeller algorithm, which uses WorldClim climate layers [95]. For each given climate variable the algorithm finds the minimum and maximum value at all sites of occurrence. The probability of occurrence is determined as: *p* = layers within min-max threshold/number of layers, e.g. [96] (Fig. 4).

## Supporting Information

Table S1 **Historical records of Lantana.** (**a**) Historical records of Lantana in Australia, (**b**) Historical records of Lantana in India (focused around Nilgiri Biosphere Reserve), (**c**) Historical records of Lantana in South Africa.
(DOC)

## References

[1] Davis MA, Chew MK, Hobbs RJ, Lugo AE, Ewel JJ (2011). Don't judge species on their origins.. Nature.

[2] Simberloff D, Alexander J, Allendorf F, Aronson J, Antunes PM (2011). Non-natives: 141 scientists object.. Nature.

[3] Alyokhin A (2011). Non-natives: Put biodiversity at risk.. Nature.

[4] Lockwood JL, Hoopes MF, Marchetti MP (2011). Non-natives: Plusses of invasion ecology.. Nature.

[5] Lerdau M, Wickham JD (2011). Non-natives: Four risk factors.. Nature.

[6] Thomas SE, Ellison CA, Spencer NR (2000). A century of classical biological control of Lantana camara: can pathogens make a significant difference?. Proceedings of the X international symposium on biological control of weeds, 4–14 July 1999.

[7] Parsons WT, Cuthbertson EG (2001). Noxious weeds of Australia, 2nd edn.

[8] Day MD, Wiley CJ, Playford J, Zalucki MP (2003). Lantana: Current management, status and future prospects. ACIAR Monograph 102.

[9] GISIN (2011). Global Invasive Species Information Network.. http://www.niiss.org/cwis438/Websites/GISINDirectory/SpeciesStatus_TopInvasives.php?WebSiteID=4.

[10] Lowe S, Browne M, Boudjelas S, De Poorter M (2000). 100 of the world's worst invasive alien species: A selection from the global invasive species database.

[11] Richardson DM, Rejmánek M (2011). Trees and shrubs as invasive alien species - A global review.. Diversity Distrib.

[12] Shaanker UR, Joseph G, Aravind NA, Kannan R, Ganeshaiah KN, Perrings C, Mooney H, Williamson M (2010). Invasive plants in tropical human-dominated landscapes: Need for an inclusive management strategy.. Bioinvasions and globalization: ecology, economics, management and policy.

[13] Pyšek P, Richardson DM (2010). Invasive species, environmental change and management, and health.. Ann Rev Env Resour.

[14] Richardson DM, Whittaker RJ (2010). Conservation biogeography – Foundations, concepts and challenges.. Diversity Distrib.

[15] Strayer DL, Eviner VT, Jeschke JM, Pace ML (2006). Understanding the long-term effects of species invasions.. Trends Ecol Evol.

[16] Essl F, Dullinger S, Rabitsch W, Hulme PE, Hülber K (2011). Socioeconomic legacy yields an invasion debt.. P Natl Acad Sci USA.

[17] Wells MJ, Stirton CH (1988). *Lantana camara*: A poisonous declared weed. Farming in South Africa. Weeds A-27.

[18] Simelane DO (2005). Biological control of Lantana camara in South Africa: Targeting a different niche with a root-feeding agent, *Longitarsus* sp.. BioControl.

[19] Queensland Government (2011). Fact sheet: Declared Class 3 Pest Plant, Lantana, *Lantana camara*.

[20] Sharma GP, Raghubanshi AS (2011). How Lantana invaded India. Current Conservation.. http://www.currentconservation.org/?p=114.

[21] Cronk QCB, Fuller JL (1995). Plant invaders.

[22] Sharma GP, Raghubanshi AS, Singh JS (2005). Lantana invasion: An overview.. Weed Biol Manag.

[23] Bailey J (1841). Plants in flower in the month of June (A list of plants flowering in the Botanical Gardens of South Australia in June 1841).. South Australian Magazine.

[24] Hough J (1829). Letters on the climate, inhabitants, productions, of the Neilgherries, or, Blue Mountains of Coimbatoor, South India.

[25] McGibbon J (1858). Catalogue of plants in the Botanic Garden, Cape Town.

[26] Johnson S (2008). Review of the declaration of Lantana species in New South Wales.

[27] Beddome RH, Griggs HB (1880). Chapter VI, Flora.. Nilagiri District Manual.

[28] Bailey FM, Tenison-Woods JE (1879). A census of the flora of Brisbane.. P Lin Soc N S W.

[29] Hooker JD (1885). Flora of British India, Vol IV.

[30] Henderson M, Anderson JG (1966). Common weeds in South Africa. Memoirs of the Botanical Survey of South Africa No. 37.

[31] Swezey OH (1916). A Natural Enemy of the Lantana.. Agric Gaz N S W.

[32] Troupe RS (1921). Silviculture of Indian trees, Vol II.

[33] Naude CP, Serfontein J (1956). Eradication of Lantana and thorn trees.. Farming in South Africa.

[34] Coode J (1930). Working plan for the deciduous forests of the Wynaad Plateau.

[35] Truman R (1968). Weed control research 1946–1967. Forestry Commission of New South Wales, Technical Paper No 17.

[36] Binns DL, Chapman WS (1992). Flora survey, Wingham management area, Port Macquarie Region, New South Wales. Forest Resources Series No 18.

[37] Henderson L (1989). Invasive alien woody plants of Natal and the north-eastern Orange Free State.. Bothalia.

[38] Muniappan R, Viraktamath CA (1986). Status of biological control of the weed, *Lantana camara* in India.. Int J Pest Manage.

[39] Haseler WH (1979). Annual Report 1978–79 Sir Alan Fletcher Research Station. Queensland, Department of Lands.. Secondary Journal, Biocontrol News and Information.

[40] Marsh EK (1978). The cultivation and management of commercial pine plantations in South Africa. Forestry and Environmental Conservation Branch Bulletin 56.

[41] Forestry Commission of New South Wales (1959–1983). Annual report of the Forestry Commission of New South Wales for the year ended 30 June, 19xx.

[42] Department of Forestry (1980). Forestry in South Africa.

[43] Cilliers CJ (1983). The weed, *Lantana camara* L., and the insect natural enemies imported for its biological control into South Africa.. J Entomol Soc S Afr.

[44] Rampati R (2006). Working Plan for the Nilgiris South Division for the period 1996–2006.

[45] Binggeli P, Goodman SM, Benstead JP (2003). Verbenaceae, *Lantana camara*, fankatavinakoho, fotatra, mandadrieko, rajejeka, radredreka, ramity. The natural history of Madagascar.

[46] Gentle CB, Duggin JA (1998). Interference of *Choricarpia leptopetala* by *Lantana camara* with nutrient enrichment in mesic forests on the central coast of NSW.. Plant Ecol.

[47] Achhireddy NR, Singh M (1984). Allelopathic Effects of Lantana (*Lantana camara*) on Milkweed vine (*Morrenia odorata*).. Weed Sci.

[48] Achhireddy NR, Singh M, Achhireddy LL, Nigg HN, Nagy S (1985). Isolation and partial characterization of phytotoxic compounds from Lantana (*Lantana camara* L.).. J Chem Ecol.

[49] Jain R, Singh M, Dezman DJ (1989). Qualitative and quantitative characterization of phenolic-compounds from Lantana (*Lantana camara*) leaves.. Weed Sci.

[50] Khan M, Srivastava SK, Jain N, Syamasundar KV, Yadav AK (2003). Chemical composition of fruit and stem essential oils of *Lantana camara* from northern India.. Flavour Frag J.

[51] Gentle CB, Duggin JA (1997). Allelopathy as a competitive strategy in persistent thickets of *Lantana camara* L. in three Australian forest communities.. Plant Ecol.

[52] Swarbrick JT, Willson BW, Hannan-Jones MA, Panetta FD, Groves RH, Shepherd RCH (1998). *Lantana camara* L. The Biology of Australian Weeds..

[53] Breman E, Bhagwat SA, Thekaekara T, Thornton TF, Willis KJ (2012). Ecosystem impacts of *Lantana camara* L. invasions.. Diversity Distrib.

[54] Henderson L (2007). Invasive, naturalized and casual alien plants in southern Africa: A summary based on the Southern African Plant Invaders Atlas (SAPIA).. Bothalia.

[55] Australian Government (2011). Weeds of national significance: Weed management guide, Lantana (*Lantana camara*).. http://www.weeds.gov.au/publications/guidelines/wons/pubs/l-camara.pdf.

[56] Batianoff GN, Butler DW (2003). Impact assessment and analysis of sixty-six priority invasive weeds in southeast Queensland.. Plant Protection Quarterly.

[57] Queensland Forest Service (1973). Annual report of the Department of Forestry for the year 1972–73.

[58] Baars JR, Neser S (1999). Past and present initiatives on the biological control of *Lantana camara* (Verbenaceae) in South Africa.. African Entomology Memoir.

[59] Love A, Babu S, Babu CR (2009). Management of Lantana, an invasive alien weed, in forest ecosystems of India.. Curr Sci.

[60] Culvenor CCJ (1985). Treatment of Lantana poisoning of cattle and sheep. In Plant toxicology.

[61] AEC Group (2007). Economic impact of lantana on the Australian grazing industry.

[62] Bailey FM (1879). On some of the introduced plants of Queensland.. Proc Linn Soc N S W.

[63] DECC (2011). *Lantana camara* - key threatening process listing. Department of Environment and Climate Change, New South Wales.. http://www.environment.nsw.gov.au/determinations/LantanaKtp.htm.

[64] Thakur ML, Ahmad M, Thakur RK (1992). Lantana weed (*Lantana camara* var. aculeata Linn.) and its possible management through natural insect pests in India.. Indian Forester.

[65] Stirton CH (1977). Some thoughts on the polyploid *Lantana camara* L, (Verbenaccae). In Proceedings of the Second National Weeds Conference, Stellenbosch, South Africa.

[66] Bernstein EM (1940). War and the pattern of business cycles.. Am Econ Rev.

[67] Knowles LCA, Knowles CM (1924). The economic development of the British overseas empire.

[68] Marais H (2001). South Africa: Limits to change? The political economy of transition.

[69] Annett S (2002). Victorian agriculture 1904–2000: Land use change or transition? In Proceedings of the Conference on Land Use Change, Department of Sustainability and Environment, August 19–20, Attwood.

[70] Goldewijk KK, Ramankutty N (2004). Land cover change over the last three centuries due to human activities: The availability of new global data sets.. GeoJournal.

[71] Francis W (1908). Madras district gazetteers: The Nilgiris.

[72] Hockings P, Hockings P (1989). The cultural ecology of the Nilgiris District.. Blue Mountains: The ethnography and biogeography of a South Indian region.

[73] ALU (2007). http://adl.brs.gov.au/mapserv/landuse/.

[74] Jha CS, Dutt CBS, Bawa KS (2000). Deforestation and land use changes in Western Ghats, India.. Curr Sci.

[75] Dukes JS, Mooney HA (1999). Does global change increase the success of invaders?. Trends Ecol Evol.

[76] Masters G, Norgrove L (2009). Climate change and Invasive alien species.. http://www.cabi.org/Uploads/File/CABi%20worldwide/Invasive%20alien%20species%20working%20paper.pdf.

[77] Fensham RJ, Fairfax RJ, Cannell RJ (1994). The invasion of *Lantana camara* L. in Forty Mile Scrub National Park, north Queensland.. Aust J Ecol.

[78] Hiremath AJ, Sundaram B (2005). The fire-Lantana cycle hypothesis in Indian forests.. Conservation and Society.

[79] Berry ZC, Wevill K, Curran TJ (2011). The invasive weed *Lantana camara* increases fire risk in dry rainforest by altering fuel beds.. Weed Res.

[80] Goulson D, Derwent LC (2004). Synergistic interactions between an exotic honeybee and an exotic weed: pollination of *Lantana camara* in Australia.. Weed Res.

[81] Feinsinger P (1987). Effects of plant species on each others pollination: Is community structure influenced?. Trends Ecol Evol.

[82] Websters (2011). Websters online dictionary: Lantana.. http://www.websters-online-dictionary.org/definitions/lantana?cx=partner-pub-0939450753529744%3Av0qd01-tdlq&cof=FORID%3A9&ie=UTF-8&q=lantana&sa=Search#906.

[83] Sharma OP, Sharma S, Pattabhi V, Mahato SB, Sharma PD (2007). A review of the hepatotoxic plant *Lantana camara*.. Crit Rev Toxicol.

[84] Greathead DJ (1968). Biological control of Lantana - a review and discussion of recent developments in East Africa.. PANS: Pest Articles & News Summaries.

[85] Ghisalberti EL (2000). *Lantana camara* L. (Verbenaceae).. Fitoterapia.

[86] Soni PL, Naithani S, Gupta PK, Bhatt A, Khullar R (2006). Utilization of economic potential of *Lantana camara*.. Indian Forester.

[87] Huntley BJ, Sandlund OT, Schei PJ, Viken A (2001). South Africa's experience regarding alien species: impacts and controls.. Invasive species and biodiversity management.

[88] Department of Primary Industries and Fisheries (2008). Biosecurity Queensland fact sheet: *Lantana camara*.

[89] Joshi AP (2002). Lantana.

[90] Singh U, Wadhwani A, Johri B (1996). Dictionary of economic plants of India.

[91] Holling CS (1978). Adaptive Environmental Assessment and Management.

[92] Sax DF, Gaines SD (2008). Species invasions and extinction: The future of native biodiversity on islands.. P Natl Acad Sci USA.

[93] Moher D, Liberati A, Tetzlaff J, Altman DG, The PRISMA Group (2009). Preferred Reporting Items for Systematic Reviews and Meta-Analyses: The PRISMA Statement.. PLoS Med.

[94] GBIF (2011). Global Biodiversity Information Facility.. http://www.gbif.org.

[95] WorldClim (2011). WorldClim – Global Climate Data.. http://www.worldclim.org.

[96] Piñeiro R, Aguilar JF, Munt DD, Feliner GN (2007). Ecology matters: Atlantic-Mediterranean disjunction in the sand-dune shrub *Armeria pungens* (Plumbaginaceae).. Mol Ecol.

[97] Henderson L (1995). Plant invaders of Southern Africa.

