# Supplementary material for: A Battle Lost? Report on Two Centuries of Invasion and Management of Lantana camara L. in Australia, India and South Africa
Source: PLoS One. 2012 Mar 5;7(3):e32407. doi: 10.1371/journal.pone.0032407 (PMC3293794; doi:10.1371/journal.pone.0032407)
Supplement: Table S1 — Historical records of Lantana. (a) Historical records of Lantana in Australia, (b) Historical records of Lantana in India (focused around Nilgiri Biosphere Reserve), (c) Historical records of Lantana in South Africa. (DOC) [file pone.0032407.s001.doc]

**A battle lost? Report on two centuries of invasion and management of *Lantana camara* L.in Australia, India and South Africa**

**Supplementary information**

**Table S1a: Historical records of Lantana in Australia**

| **Year** | **Report** |
| --- | --- |
| 1841 | Lantana introduced to the old Botanic Gardens in Adelaide, South Australia. There have been multiple introductions since, mainly in New South Wales and Queensland (Bailey, 1841; Bailey, 1879; DECC, 2011). |
| 1843 | Cultivated plants are known to have been grown in New South Wales by John Macarthur at Camden Park (Anonymous, 1843; Michael, 1972; Bailey, 1879; DECC, 2011). |
| c.1844 | Present in eastern Australia (GISD, 2011a). |
| 1840s | Common pink Lantana reputed to have been introduced to NSW from SA by Major Innes (KR Green, Department of Agriculture NSW pers com 1966). Cultivated extensively, probably as an ornamental hedge, on his property in Port Macquarie (Smith and Smith, 1982). |
| 1850s | Lantana spread to south eastern Australia, recorded as naturalised (Johnson, 2008) (GISD, 2011b). |
| 1861 | Reported in Brisbane (Johnson, 2008). |
| 1869 | In the Hastings and Clarence catchments of NSW in the late 1860’s (Swarbrick 1986). Earliest recorded specimen of ‘Common Pink’ variety in Australia collected near Hastings River NSW by Dr Beckler, housed at National Herbarium of Victoria (Smith and Smith, 1982). |
| 1870 | Lantana introduced to east coast of Australia (map in Stirton 1977). |
| 1879 | Reported as a "most troublesome weed" in Queensland and abundant around Port Jackson, New South Wales (Bailey, 1879; Bailey and Tenison-Woods, 1879). |
| 1895 | Listed as one of the ten worst weeds in NSW in 1895 (Maiden, 1895) and 1920 (Maiden, 1920). |
| 1914 | First biological control programme for Lantana in Australia (Department of Primary Industries and Fisheries, 2008). |
| 1916 | Considering biological control options based on insects used in Hawaii (Swezey, 1916). |
| 1919 | Euxoa (Agrotis) radians, Guen, a maize pest that also attacks Lantana (Beard, 1919) |
| 1920 | Lantana more or less eradicated from Biggenden and Amamoor reserves. Experiments in Goodnight Scrub State Forest on destruction of Lantana by Lantana fly failed (Queensland Department of Public Lands, 1920). |
| 1921 | Lantana is spreading and shows an inimical effect in respect to the reproduction of jungle spp (Queensland Department of Public Lands, 1921). |
| 1921 | One of the most prevalent noxious weeds of reserves and vacant crown lands of north Queensland. This pest is spreading at an alarming rate, having made unheard-of progress since its first appearance in the Carins district. Lantana is at present confined to an area <15 miles from the coast, where it is making a rapid spread. All forest officers asked to report immediately any sightings and to eradicate. Now it exists in large bodies in the Redlynch district, up the Cairns Range to Kuranda and Oaklands (District Forester Fraser in Queensland Department of Public Lands, 1921). |
| 1921-1980 | Information on the control of Lantana in Queensland forestry plots using variety of methods with costs (Queensland Department of Public Lands, 1921; Queensland Department of Forestry, 1980). |
| 1935 | The Lantana bug (TeleonemiascrupulosaStal.) a native of Central America, was introduced to Australia from Fiji (Fyfe, 1937) in (Cashmore and Campbell, 1946). |
| 1936 | Comprehensive report on weed problems in Australia (Currie, 1936). |
| 1942 | *L. camara*, and *L. montevidensis* (Spreng.) Briq. [which replaces *L. sellowiana* Link & Otto (Lantana creeping)] listed as weed species (CSIRO, 1942). |
| 1946 | *L. camara* weed of tropical and sub-tropical coastal areas of Queensland and NSW. Colonizes post fire or invades cleared grazing areas and forest plantations. Two releases of the Lantana bug *Teleonemia scrupulosa* Stal. in 1936 and 1939 near Atherton, Queensland led to large populations and defoliation of many bushes of Lantana (Currie and Fyfe, 1939). Further releases were made and the bug is now widespread in Lantana-infested regions north of Townsville. Colonies of bugs also established south of Townsville, but effects less spectacular. Numerous attempts have been made to establish colonies in coastal districts of northern NSW but no success as yet - probably a climate limitation (Cashmore and Campbell, 1946). |
| 1946-1967 | Review of chemical control of Lantana camara in forest plantations. In Boambee State Forest controlled Lantana in early trials with 2.4-D esters, enough to allow tree establishment. In Mt Pikapene State Forest experiments with weedicides along fire breaks saved 70-80% costs over chipping, extended into forests by 1954 and report 95% Lantana. Total cost $28.05 per acre ($40 for chipping and re-growth by April). 2.4-D amine salt better than esters as leaf drop slower. Used in aerial spray experiments in 1960 – sparse plants died or knocked back, dense stands recovered in 6 months to original levels, pines increased in height and crown size in sprayed areas (Truman, 1968). |
| 1952-1958 | Burn trials for control of Lantana in Maryborough district Queensland, found that annual burns necessary to control the weed, but that this didn’t necessarily result in a decline in Lantana populations, and can be detrimental to growing stock under certain conditions e.g. dense infestations if don’t burn get dense infestations of Lantana and other weeds. Between 1952-8 Lantana more than doubles in height and shows ‘a remarkable increase’ in area covered in un-burnt plots. In burnt areas the number of plants present shows a slight increase but average height is lower in 1958 and the increase in area covered is slight. Seedlings are killed by fire but enough survive to account for greater stocking of Lantana in burnt plots. Overall burning restricts the spread of Lantana by preventing development of large clumps (Henry, 1960). |
| 1959 | Lantana is a pest in many forest areas along the north coast of NSW. Chemical and mechanical methods of control are costly and of doubtful value in forest country. Looking to Queensland for insects for biological control (Forestry Commission of New South Wales, 1959). |
| 1959-1983 | Breeding (and later release) programmes for biocontrol, reports on insects used and their impact (Forestry Commission of New South Wales, 1969 through 1986; Forestry Commission of New South Wales, 1959 through 1983). |
| 1961 | Preliminary survey of extent and severity Lantana infestation on State Forests has been initiated (Forestry Commission of New South Wales, 1962). |
| 1965 | Speed with which Lantana encroaches onto roadways and cleared areas is well known. Control by slashing or mechanical clearing is both slow and expensive, while biological control methods are not yet available (Forrest and Richardson, 1965). Gives information on the most effective herbicides and their application. |
| 1966 | Lantana camara serious pest in Queensland and NSW, set up trials in Hawaii to see if species that attach Lantana also attack Australian plants of economic importance (CSIRO, 1966). |
| 1967 | c.18 varieties of L. camara identified in taxonomic study (Forestry Commission of New South Wales, 1968). |
| 1968 | Leaf mining beetles (*Octotoma scabripennis* and *Uroplata girardi*) first collected from Queensland for release in NSW (Forestry Commission of New South Wales, 1968). |
| 1968 | Trials of different weed killers on Lantana, found to be more cost effective than mechanical control (Truman, 1968). |
| 1969 | Two forms of Lantana, one wild form, naturalised, run out from cultivation, troublesome weed in a few areas, the other a garden form (Henderson, 1969). |
| 1970 | 29 taxa of *Lantana camara* recognised in Australia. Occur from Cooktown in Queensland to Ulladulla in NSW, extend west to Great Dividing Range. 19 taxa are sufficiently common to be of economic importance either as weeds or poisonous plants, remaining 10 sparingly naturalised and not of economic significance at present. 14 are toxic, three non-toxic and the rest unknown (Smith and Smith, 1982). |
| 1972 | ‘Biological control of Lantana remains the largest single Forest Entomology project’ (Forestry Commission of New South Wales, 1973). |
| 1973 | Lantana is the most serious weed in Hoop pine plantations and requires regular tending in plantations of all ages, not such a problem in exotic pine, Lantana can establish where fire excluded but slower and if monitor can control (e.g. by slash and burn) (Queensland Forest Service, 1973). |
| 1973 | Department with particular interest in biological control of Lantana as cost of controlling in plantations and along roadside together with loss of revenue due to increase in harvesting costs and lower productivity in native forests would be about $1M/yr (Queensland Forest Service, 1973). |
| 1973 | c.30 varieties of L. camara in Australia, of the nine at pest proportions eight are toxic to livestock, working on chemical constituents of Lantana to better understand biological control and effect on livestock (CSIRO, 1973). |
| 1977 | Lantana main weed problem in older exotic pine plantations, but prescribed burning (now more widely practiced) should control this (Queensland Department of Forestry, 1977). |
| 1977-1979 | Biocontrol having effect in Queensland, though only effective insects are the leaf miners (Department of Forestry Queensland, 1977; Department of Forestry Queensland, 1979). |
| 1979 | The spread of *Lantana camara* is being controlled in some areas by *Octotoma scabripennis* and *Europlata girardi* (Haseler, 1979). In general optimism about biocontrol from late 1960s through 1970s, but keep having failures, and those that do succeed don’t do as well as hoped (Forestry Commission of NSW, various years). |
| 1983 | Biological control of Lantana by leaf miners at sites north of Coffs Harbour generally successful, reduce vigour and aggressiveness of Lantana allowing other plants to compete more successfully (Forestry Commission of New South Wales, 1983). |
| 1992 | Invasion of plantations by exotic weeds seen as a threat (Resource Assessment Commission, 1992). |
| 1992 | In the Wingham management area, Port Macquarie Region, New South Wales *L. camara* naturalised alien: found in 2/127 plots. Occurs in localised areas with heavy disturbance e.g. log dumps, and only persist where disturbance regular e.g. roadsides, recreational areas that are heavily used or regularly maintained (Binns and Chapman, 1992). |
| 1993 | *L. camara* is a declared Noxious Weed under the NSW Noxious Weeds Act 1993 |
| 1993 | *L. camara* prevalent in most vegetation types of coastal forest NSW in Kempsey and Wauchope management areas (27/200 plots), not in rainforest or heathland. Naturalised, frequent at altitudes below about 500m, especially on disturbed sites, can dominate understory (Binns and Chapman, 1993). |
| 1994 | *L. camara* problem in Grafton Forest management area, found in 48/182 plots, control measures discussed (Moore and Floyd, 1994; Margules Groome Pӧyry Pty Ltd, 1994). |
| 1998 | Plan for afforestation of farm lands in NSW recommends that all Lantana should be killed, glycophosphate effective, and left in place as important habitat for small insectivorous birds in the absence of native vegetation, also acts as physical barrier to keep livestock out of re-vegetated areas (Brouwer, 1998). |
| 2002 | All Lantana species are declared Class 3 plants under the Land Protection (Pest and Stock Route Management) Act 2002. Lantana species cannot be sold or distributed and landholders may be required to control these plants if they pose a threat to an environmentally significant area (Department of Primary Industries and Fisheries, 2008). |
| 2003 | Lantana ranked the highest in invasive species assessment for Queensland (Batianoff and Butler, 2003). |
| 2004 | Lantana control manual issued by Australian Government, 88 pages practical information available from Weeds Australia website (Australian Government, 2004). |
| 2008 | Currently, Lantana covers more than 4 million ha of the east coast from southern New South Wales to far north Queensland. Small infestations of Lantana have also been found in central west Queensland, the Northern Territory, Western Australia, South Australia and Victoria. Efforts are under way to control these (Department of Primary Industries and Fisheries, 2008). |
| 2009 | To combat the worst impacts of Lantana on the environment, a national Plan to Protect Environmental Assets from Lantana was instituted with funding from the Australian Federal Government and support from various state agencies, regional groups and private individuals (National Lantana Management Group, 2009). Environmental assets are defined here as native species, populations, Regional Ecosystems and ecological communities. |
| 2011 | The mechanisms behind the impact of Lantana are yet to be fully investigated. Data on the impact of certain management actions (such as the use of herbicide and fire) on threatened species, or more generally on native species is also limited. Such information is necessary to manage and better protect threatened biodiversity. Rigorous examination needs to be undertaken by researchers into the effects of Lantana as well as management on threatened species (New South Wales Government, 2011). |
| 2011 | *L. camara* occurs along the east coast of Australia, from southern NSW to Cape York in Queensland, and from sea-level up to 600 m altitude, or less commonly to 1000 m. Invaded at least 4 million hectares, mainly in NSW and Queensland. In NSW most infestations are north of the Clyde River, with occasional mostly small occurrences from there south to the Victorian border; one of these however, at Mt Dromedary (Gulaga National Park and surrounds), is large. *L. camara* is also naturalised in the Northern Territory, South Australia and Western Australia, on Lord Howe and Norfolk Islands, and (apparently marginally) in Victoria. *L. camara* is not known to yet be naturalised in Tasmania, but its importation to that State is prohibited and a Statutory Weed Management Plan has been prepared (DECC, 2011). |

***References***

Anonymous (1843) Catalogue of plants cultivated at Camden, New South Wales. Sydney.

Australian Government (2004) Lantana, current management and control options for Lantana (*Lantana camara*) in Australia. Weeds of National Significance. The National Heritage Trust, the Department of Natural Resources, Mines and Energy, Queensland, and New South Wales Department of Agriculture.

Bailey FM (1879) On some of the introduced plants of Queensland. Proceedings of the Linnean Society of New South Wales 4: 26-36.

Bailey FM Tenison-Woods JE (1879) A cencus of the flora of Brisbane. Proceedings of the Linnean Society of New South Wales 4: 137-204.

Bailey J (1841) Plants in flower in the month of June.(A list of plants flowering in the Botanical Gardens of South Australia in June 1841). South Australian Magazine 1: 30.

Batianoff GN Butler DW (2003) Impact assessment and analysis of sixty-six priority invasive weeds in southeast Queensland. Plant Protection Quarterly 18: 11-17.

Beard JS (1919) The Insect Pests of Maize. Agriculture Gazette N.S.W. 30: 196-202.

Binns DL Chapman WS (1992) Flora survey, Wingham management area, Port Macquarie Region, New South Wales. Forest Resources Series No 18.Beecroft, NSW, Forestry Commission of NSW, Research Division.

Binns DL Chapman WS (1993) Flora survey, Kempsey and Wauchope management areas, Central Region, New South Wales. Forest Resources Series No 24.Beecroft, NSW, Forestry Commission of NSW, Research Division.

Brouwer D (1998) Plan for trees: a guide to farm revegetation on the coast and tablelands. NSW Agriculture, Tocal, Australia.

Cashmore AB Campbell TG (1946) The weeds problem in Australia: a review. Journal for the Council for Scientific and Industrial Research 19.

CSIRO (1942) Standardized plant names. A list of standard common names for the more important Australian grasses, other pasture plants and weeds. Bulletin No 156. Melbourne, Council for Scientific and Industrial Research, Commonwealth of Australia.

CSIRO (1966) CSIRO Annual Report 1965-66. Melbourne, Commonwealth Scientific and Industrial Research Orgnaisation.

CSIRO (1973) Division of Applied Chemistry, Annual Report 1972-3. Melbourne, Commonwealth Scientific and Industrial Research Organisation, Australia.

Currie GA (1936) A report on a survey of weed problems in Australia. Pamphlet 60.Council for Scientific and Industrial Research, Australia.

DECC (2011) Lantana camara - key threatening process listing. Department of Environment and Climate Change, New South Wales, http://www.environment.nsw.gov.au/determinations/LantanaKtp.htm Accessed 15.3.11.

Department of Forestry Queensland (1977) Research report 1977. Report of research activities.Division of Technical Services, Queensland Department of Forestry.

Department of Forestry Queensland (1979) Research report 1979. Report of research activities for 1978, 1979.Division of Technical Services, Queensland Department of Forestry.

Department of Primary Industries and Fisheries (2008) Biosecurity Queensland fact sheet: *Lantana camara*. Land Protection (Invasive Plants and Animals), Queensland, Australia.

Forestry Commission of New South Wales (1959) Report of the Forestry Commission of New South Wales for the year ended 30 June, 1958.

Forestry Commission of New South Wales (1962) Report of the Forestry Commission of New South Wales for the year ended 30 June, 1961.

Forestry Commission of New South Wales (1968) Report of the Forestry Commission of New South Wales for the year ended 30 June, 1967.

Forestry Commission of New South Wales (1969) Research report 1968. Sydney, Australia.

Forestry Commission of New South Wales (1973) Report of the Forestry Commission of New South Wales for the year ended 30 June, 1973.

Forestry Commission of New South Wales (1983) Research report 1981 & 1982. Sydney, Australia.

Forrest WG Richardson RR (1965) Chemical control of forest weeds.Research Note 16.Forestry Commission of New South Wales.

Fyfe RV (1937) The lantana bug, Teleonemialantanae Distant. . Journal of the Council for Science and Industry Research, Australia 15.

GISD (2011a) Lantana in eastern Australia.Global Invasive Species Database. http://www.issg.org/database/species/distribution_detail.asp?si=56&di=16221&pc=*&lang=EN.

GISD (2011b) Lantana in south eastern Australia.Global Invasive Species Database. http://www.issg.org/database/species/distribution_detail.asp?si=56&di=16228&pc=*&lang=EN.

Haseler WH (1979) Annual Report 1978-79 Sir Alan Fletcher Research Station. Queensland, Department of Lands. Secondary Journal, Biocontrol News and Information 1: 317.

Henderson RJF (1969) A cytological study of Lantana montevidensis (Spreng.)Briq.in Queensland. Contributions from the Queensland herbarium.Brisbane, Queensland Herbarium, Department of Primary Industries.

Henry NB (1960) Complete protection versus prescribed burning in the Maryborough hardwoods. Research Note No 13 (311 - Bn 072).Brisbane, Queensland Department of Forests.

Johnson S (2008) Review of the declaration of Lantana species in New South Wales. Orange, New South Wales: Department of Primary Industries.

Maiden JH (1895) The weeds of New South Wales with suggested measures, legislative or otherwise, for their destruction. Part 1 General. Agricultural Gazette of New South Wales 6: 152-158.

Maiden JH (1920) The weeds of New South Wales. Part 1. Sydney: Government Printer.

MargulesGroomePӧyry Pty Ltd (1994) Environmental impact statement Grafton management area.State Forests of New South Wales.

Michael PW (1972) The weeds themselves - early history and identification. Proceedings of the Weed Society of New South Wales 5: 3-18.

Moore DM Floyd AG (1994) A description of the flora and an assessment of impacts of the proposed forestry operations in the Grafton Forest management area.Grafton management area EIS supporting document No. 2.State Forests of New South Wales.

National Lantana Management Group (2009) Environmental impacts of Lantana in Australia. http://www.weeds.org.au/WoNS/lantana/docs/67_Environmental_impacts2.pdf Accessed 11.3.11.

New South Wales Government (2011) Pests and Weeds: Lantana. http://www.environment.nsw.gov.au/lantanaplan/research.htm. Office of Environment and Heritage, NSW Government.

Queensland Department of Forestry (1977) Annual report 1976-77. Brisbane.

Queensland Department of Forestry (1980) Forestry annual report 1979-80. Brisbane.

Queensland Department of Public Lands (1920) Annual report of the Director of Forests for the year ended 30th June, 1920.

Queensland Department of Public Lands (1921) Annual report of the Director of Forests for the year ended 30th June, 1921.

Queensland Forest Service (1973) Annual report of the Department of Forestry for the year 1972-73. Brisbane.

Resource Assessment Commission (1992) Forest and timber inquiry final report. Volume 2a. Canberra.

Smith LS Smith DA (1982) The naturalised Lantana camara complex in eastern Australia. Queensland Botany Bulletin 1: 1-26.

Swezey OH (1916) A Natural Enemy of the Lantana. Agric. Gaz. N.S.W..1916, November. 27: part 11, 798p.

Truman R (1968) Weed control research 1946-1967.Forestry Commission of New South Wales, Technical Paper No 17.New South Wales Department of Conservation.

Forestry Commission of New South Wales (1959) Report of the Forestry Commission of New South Wales for the year ended 30 June, 1958.

Forestry Commission of New South Wales (1960) Report of the Forestry Commission of New South Wales for the year ended 30 June, 1959.

Forestry Commission of New South Wales (1961) Report of the Forestry Commission of New South Wales for the year ended 30 June, 1960.

Forestry Commission of New South Wales (1962) Report of the Forestry Commission of New South Wales for the year ended 30 June, 1961.

Forestry Commission of New South Wales (1967) Report of the Forestry Commission of New South Wales for the year ended 30 June, 1966.

Forestry Commission of New South Wales (1968) Report of the Forestry Commission of New South Wales for the year ended 30 June, 1967.

Forestry Commission of New South Wales (1969) Research report 1968. Sydney, Australia.

Forestry Commission of New South Wales (1970) Research report 1969. Sydney, Australia.

Forestry Commission of New South Wales (1971) Report of the Forestry Commission of New South Wales for the year ended 30 June, 1970.

Forestry Commission of New South Wales (1972) Report of the Forestry Commission of New South Wales for the year ended 30 June, 1971.

Forestry Commission of New South Wales (1973) Report of the Forestry Commission of New South Wales for the year ended 30 June, 1972.

Forestry Commission of New South Wales (1973) Report of the Forestry Commission of New South Wales for the year ended 30 June, 1973.

Forestry Commission of New South Wales (1973) Research report 1971 & 1972. Sydney, Australia.

Forestry Commission of New South Wales (1974) Report of the Forestry Commission of New South Wales for the year ended 30 June, 1974.

Forestry Commission of New South Wales (1975) Report of the Forestry Commission of New South Wales for the year ended 30 June, 1975.

Forestry Commission of New South Wales (1975) Research report 1973 & 1974. Sydney, Australia.

Forestry Commission of New South Wales (1976) Report of the Forestry Commission of New South Wales for the year ended 30 June, 1976.

Forestry Commission of New South Wales (1977) Report of the Forestry Commission of New South Wales for the year ended 30 June, 1977.

Forestry Commission of New South Wales (1978) Report of the Forestry Commission of New South Wales for the year ended 30 June, 1978.

Forestry Commission of New South Wales (1978) Research report 1975 & 1976. Sydney, Australia.

Forestry Commission of New South Wales (1979) Report of the Forestry Commission of New South Wales for the year ended 30 June, 1979.

Forestry Commission of New South Wales (1980) Research report 1977 & 1978. Sydney, Australia.

Forestry Commission of New South Wales (1981) Report of the Forestry Commission of New South Wales for the year ended 30 June, 1981.

Forestry Commission of New South Wales (1981) Research report 1979 & 1980. Sydney, Australia.

Forestry Commission of New South Wales (1982) Report of the Forestry Commission of New South Wales for the year ended 30 June, 1982.

Forestry Commission of New South Wales (1983) Report of the Forestry Commission of New South Wales for the year ended 30 June, 1983.

Forestry Commission of New South Wales (1983) Research report 1981 & 1982. Sydney, Australia.

Forestry Commission of New South Wales (1986) Research report 1983 & 1984. Sydney, Australia.

Queensland Department of Forestry (1977) Annual report 1976-77. Brisbane.

Queensland Department of Forestry (1980) Forestry annual report 1979-80. Brisbane.

Queensland Department of Public Lands (1920) Annual report of the Director of Forests for the year ended 30th June, 1920.

Queensland Department of Public Lands (1921) Annual report of the Director of Forests for the year ended 30th June, 1921.

Queensland Forest Service (1921) Annual report of the Director of Forests for the six months ended 31st December, 1921.

Queensland Forest Service (1922) Annual report of the Director of Forests for the year ended 31st December, 1922.

Queensland Forest Service (1923) Annual report of the Director of Forests for the year ended 31st December, 1923.

Queensland Forest Service (1924) Report of the Provisional Forestry Board for the year ended 31st December, 1924.

Queensland Forest Service (1926) Report of the Provisional Forestry Board for the year ended 30th June, 1926. Brisbane, Deparment of Public Lands.

Queensland Forest Service (1927) Report of the Provisional Forestry Board for the year ended 30th June, 1927. Brisbane, Deparment of Public Lands.

Queensland Forest Service (1928) Report of the Provisional Forestry Board for the year ended 30th June, 1928. Brisbane, Deparment of Public Lands.

Queensland Forest Service (1929) Report of the Provisional Forestry Board for the year ended 30th June, 1929. Brisbane, Deparment of Public Lands.

Queensland Forest Service (1930) Report of the Provisional Forestry Board for the year ended 30th June, 1930. Brisbane, Deparment of Public Lands.

Queensland Forest Service (1931) Report of the Provisional Forestry Board for the year ended 30th June, 1931. Brisbane, Deparment of Public Lands.

Queensland Forest Service (1932) Report of the Provisional Forestry Board for the year ended 30th June, 1932. Brisbane, Deparment of Public Lands.

Queensland Forest Service (1943) Report of the Director of Forests for the year ended 30th June, 1943. Brisbane, Deparment of Public Lands.

Queensland Forest Service (1944) Report of the Director of Forests for the year ended 30th June, 1944. Brisbane, Deparment of Public Lands.

Queensland Forest Service (1945) Report of the Director of Forests for the year ended 30th June, 1945. Brisbane, Deparment of Public Lands.

Queensland Forest Service (1967) Annual report of the Department of Forestry for the year 1966-67. Brisbane.

Queensland Forest Service (1968) Annual report of the Department of Forestry for the year 1967-68. Brisbane.

Queensland Forest Service (1973) Annual report of the Department of Forestry for the year 1972-73. Brisbane.

Queensland Forest Service (1974) Annual report of the Department of Forestry for the year 1973-74. Brisbane.

**Table S1b: Historical records of Lantana in India (focused around Nilgiri Biosphere Reserve)**

| **Year** | **Report** |
| --- | --- |
| 1809 | First introduced in India in Calcutta gardens. |
| 1829 | First mentions of Lantana, in 'letters on the climate, inhabitants, productions &c. &c. of the Neilgherries'. Not a comprehensive study, and based on casual observations around the area. Two species mentioned, probably in gardens. (Hough, 1829:118).  (Assuming location to be 'Stone House', Ooty, from where to took his morning walks) |
| 1870 | Richter (1870) reports that “It [American Aloe (Agave americana)] has been superseded, however, by the Lantana Shrub (Lantana Aculeata) which within a few years has spread over the whole of coorg... when kept within proper bounds makes an excellent fence. But this plant, whose vitality is most obstinate, threatens to overrun many a tract of land in Coorg and elsewhere that may be far more profitably occupied.” |
| 1880 | Lantana mentioned as present in the region, but no hint of it as a problem, despite detailed descriptions of agriculture and and wild flora of the region. The first Gazetteer of the Nilgiris (Beddome 1880). |
| 1885 | description of Lantana being invasive, though not in the Nilgiris: “an American plant, has run wild in India, especially in the W. Deccan Peninsula and Ceylon.” Flora of British India, (Hooker 1885). |
| 1906 | Lantana “has spread with extraordinary vigour in Ceylon and the peninsula [of India]..in deciduous forests it is a most troublesome weed.. and ..is considered by some to be the cause of the spike disease of sandalwood in Coorg.” The first mention of it as a problem, though not explicitly in the Nilgiris. (Brandis 1906). |
| 1919 | Very good one document. Pest and spreading, Needs to be managed very seriously (Wood 1919). |
| 1921 | Recognition of the severity of the problem “..wherever conditions are at all favourable to its growth it spreads with alarming rapidity, and areas which in one year may contain only a few bushes sparsely dotted about will often become covered with a dense impenetrable mass of Lantana within the space of a few years. Grazing grounds get ruined in a short space of time, since the dense growth not only prevents the access of cattle, but also kills out grass.” 'Troublesome' in the Nilgiris, and details about its control (in Kodagu/Coorg) are described (Troupe 1921). Also in Nilambur. |
| 1924 | Mr Tiremen 'prescribed Lantana eradication from the infested areas near Benne and Mudumalai. This work was done for several years, though there is very little to show for it, these localities being as badly infested with Lantana as ever.' (Ranganathan 1941, but the Lantana was removed in 1924) |
| 1925 | W. Deccan and lower hills of Western Ghats, upto perhaps 6000 ft., especially common in Coorg and Wynaad, introduced from tropical America and run wild. The plant is most troublesome and measures for its destruction are often necessary though difficult (Gamble 1925) |
| 1930 | Present in Wynaad Plateau. Removed in parts. Prevents natural regeneration, but is good for soil, can be completely removed later if required. (Coode 1930) |
| 1941 | Described as being a problem - taking over the under story, had 'spread rapidly over the ground in recent years.' - in deciduous forests and teak plantations of Mudumalai, Benne and Thepakadu. (Ranganathan 1941) |
| 1942 | Problem in Wyanaad region and impedes growth of forests, but can be managed, 'Lantana prevails widely in the older plantations, but is under control in the recent ones.' and 'adds humus to the soil, however'. Lantana should be uprooted wherever it may occur inside the regeneration area at any time. (Ayyar 1942) |
| 1957 | Lantana had taken possession of the ground in dense impenetrable thickets in the young teak plantations affecting the rate of growth of teak in its early stages and also adding to the risk of fire during hot weather. Some teak is completely smothered by it and also associated with spike in sandalwood. Management '..will also include uprootal of lantana over the entire plantation area.' (Jeyadev 1957) |
| 1966 | Mettupalayam/Bhavani ranges. Lantana is a 'serious pest in all worked coupes of the fuel series and in the neighbourhood of Irular settlements. The dense growth of this obnoxious species often inhibits natural regeneration of valuable species, particularly sandal.' All Lantana must be uprooted in sandal working circles, and it is 'imperative to restock areas that have been felled else they will be overtaken by dense growth of noxious weeds like Lantana'. Problem managed (Wilson 1966) |
| 1977 | 'Lantana is a serious pest, in all working coupes; It rapidly invades places where the canopy is open and forms as impenetrable mass in two or three years. Lantana suppressed seedling regeneration of many tree species and is the most troublesome weed in the teak areas. Uprooting and burning is the only method by which Lantana can be kept under check in the regeneration areas but this is far from being an economic proposition. Greatest care must be taken in thinning young teak plantations to ensure that the canopy is not opened too much. V Jayaraman WP for Coimbatore cenral Forest Division (Jayaraman, 1977) |
| 1986 | Biocontrol (management) but still spreading in both north and south india (Muniappan and Viraktamath 1986) |
| 1987 | Lantana and Eupatorium are seen in patches especially in openings and they are causing a lot of problems for younger regeneration. (Akbar Sha 1987) Working Plan for Kodagu Forest Division |
| 1989 | “None is as abundant and Lantana, introduced from South America. It became a pest throughout much of India in the first half of the last century. Dispersed by birds, it is a wayside weed forming dense thickets mainly on poor, eroded soils. (Lengerke and Blasco, 1989) |
| 2000 | Is being managed, but partially beneficial to wildlife also (Kantharaju 2000) Management Plan for Bandipur |
| 2007 | Both good and bad so need to be managed within limits. Mudumalai Management Plan. (Dogra 2007). |
| 2009 | Spreading in spite of all controls. Review paper. For all of India, so assumed to be central/south India (Love et al. 2009). |

***Reference***

Hough, James. 1829. Letters on the climate, inhabitants, productions &c. &c. of the Neilgherries, or blue mountains of Coimbatoor, South India. P118.

Richter, G. 1870. The Gazeteer of Coorg. Mysore. Government Press.

Beddome, R. H. 1880. Chapter VI, Flora.In Griggs,H. B. (Ed.)Nilagiri District Manual.

Hooker, J.D. 1885 Flora of British India.Vol IV p562. London

Brandis, Dietrich. 1906. Indian Trees. P502. London

Wood, H.F.A. 1919. Working Plan fot the South Coimbatore Forest Division.Government Press. Madras

Troupe, R.S. 1921.Silviculture of Indian trees.Vol II p 780.Oxford University Press.

Ranganathan, C.R. 1941 Working plan for the Nilgiris Forest Division.Government Press. Madras

Gamble, J.S. 1915-1925. Flora of the presidency of Madras.Vol II. P 1068.

Coode, J. 1930 Working plan for the deciduous forests of the Wynaad Plateau.Government Press. Madras

Ranganathan, C.R. 1941 Working plan for the Nilgiris Forest Division.Government Press. Madras.

Ayyar, Venkateshwara T.V. 1942 Revised working plan for the Wyanaad division. Government Press. Madras

Jayadev, T. 1957. Working plan for the Nilgiris Forest Division.Government Press. Madras.

Wilson, J. 1966. Working plan for the Mettupalayam and Bhavani ranges of Coimbatore Central division. Government Press. Madras.

Jayaraman, V. 1977 Working plan for the Coimbatore central forest division.1972-72 to 1981-82.Government Press. Madras.

Muniappan, R. and Viraktamath, C. A.(1986) 'Status of biological control of the weed, Lantana camarain India', International Journal of Pest Management, 32: 1, 40 — 42

Akbar Sha, A. 1987. Working plan for Kodagu Forest Division .1987-1996.

Lengerke, H. J. V. and Francois Blasco, 1989.The Nilgiri Environment. In (eds) Hockings, Paul. Blue mountains, the ethnography and biogeography of a south Indian region.Delhi; New York: Oxford University Press.

Kantharaju, H.C. 2000. Management Plan for the Bandipur Tiger Reserve.Karnataka Forest Department.

Dogra, Rakesh Kumar, 2007. Management Plan for the Mudumalai National Park and Wildlife Sanctuary.Tamilnadu Forest Department.

Love, Amit, Suresh Babu and C. R. Babu. 2009. Management of Lantana, an invasive alien weed, in forest ecosystems of India. Current Science, Vol. 97, No. 10

**Table S1c: Historical records of Lantana in South Africa**

| **Year** | **Report** |
| --- | --- |
| 1858 | *Lantana aculeata*, *L. alba*, *L. selowii* and *L. trifoliate* introduced from Europe into Cape Town (McGibbon, 1858) in (Stirton, 1977) |
| 1883 | *Lantana aculeate* and *L. mutabilis* introduced to Natal from Europe (Wood, 1883) in Stirton 1977 |
| 1910 | *Lantana camara* introduced to Natal from Mauritus (Wood, 1910) in Stirton 1977 Unrecorded introductions from other parts of the world, including the West Indian Islands, Central America, Mexico and South America (Howard, 1970). |
| 1882-1962 | No mention of Lantana in Forestry and Environmental Conservation Branch Annual Reports, ‘Report of the Conservators of Forests’ for the Cape Region (also called the Annual Report for the Department of Forestry) |
| 1902-10 | No mention of Lantana in Transvaal Agricultural Journal (other weeds are included) |
| 1937 | Lists *Lantana camara* as a proclaimed noxious weed throughout the whole of South Africa under the Weeds Act (No 42, 1937), compulsory eradication by the occupier/owner of property where they are growing after the required notice has been served (Henderson & Anderson, 1966) |
| 1943 | Noted as having widely spread along Natal coast and caused poisoning in dairy cattle (Ritchie, 1943) |
| 1950 | *Lantana camara*, originally a cultivated ornamental shrub, but now a serious pest 'escape' in Natal. It smothers indigenous bush, encroaches upon veld and is poisonous to stock. Mechanical eradication has proved simple and effective, but is costly. An effective spray is 2, 4, 5-T, but this weed-killer is not yet easily obtainable in the open market. L. camara has been proclaimed a weed in Natal, and, if it spreads, the interdict may be extended to the whole Union. (Louw, 1950). |
| 1951 | Mentions *Lantana camara* as an exotic as a garden escape near Melelane (Codd, 1951). |
| 1956 | Paraffin and dieseline solutions of 2, 4, 5-T (3 1/5 oz. amyl ester in 2 gal. solvent) gave satisfactory control of Lantana (Naude&Serfontein, 1956) |
| 1960-1963 | Work on the biological control of *Lantana camara* in South Africa began in with the introduction of insects from Hawaii and Trinidad, and was resumed in 1968 with the importation and screening of possible control agents from Australia (Cilliers, 1977; Pass & Heath, 1977). |
| 1962 | Lantana covered 25-30,000 morgen in SA, 80% in Natal, 14% in Transvaal and 6% in Eastern Cape (Marr, 1964). |
| 1962 | Lantana hacked out of many plantations (Dept Forestry Annual Report, 1962) |
| 1963/4 | ‘Lantana spp were hacked out in the Transkei, particularly at the Ibeka, Kentani and Manubi plantations and on more than 189 morgen (0.86 hectare) of the Bultfontein plantation in the Eastern Transvaal Region’ (Department of Forestry, various) |
| 1964/5 | ‘In the Eastern Transvaal Region Lantana and Solonaceae were eradicated in the Bergvleit Plantation’ (Department of Forestry, various). |
| 1966 | ‘It [Lantana] has spread alarmingly in Natal and the eastern Cape Province, especially in coastal areas as well as in tropical Africa. Birds eat the fruits and so spread the seeds from garden to veld. In the Transvaal the weed is spreading. All cultivated Lantanas are considered to be varieties or forms of L. camara with the exception of L. montevidensis... The latter has not been known to spread from cultivation and may therefore be left in gardens.’ (Henderson & Anderson, 1966) |
| 1966/7 | ‘Brambles, Mauritius thorn, bug-tree and Lantana are causing trouble in the Eastern Transvaal Region.’ |
| 1967 | *Lantana camara* (and *Eupatorium odoratum*) is spreading in the Coast Lowlands and lower Coast Hinterland Regions (study from Tugela river basin) (Edwards, 1967). |
| 1971/2 | Southern Transvaal region, Lantana is spreading on private ground near Elandshoek and along the Pretoria-Nelspruit road. Eastern Transvaal Region, Lantana which occurs at Ceylon and Tweefontein is uprooted wherever found. |
| 1973/4 | Eastern Cape Region – ‘Jointed cactus and Lantana infestations were eradicated in the Mount Coke State Forest’. Northern Transvaal Region – ‘Mauritius thorn, American bramble, Lantana and *Acacia ataxacantha* are the most serious intruder weeds in this area. Weedicides have not been effective and although slashing is undertaken for up to three years in young plantation stands, this can only be regarded as an operation designed to eliminate early competition.’ Table 3.5 outlines control measures for invader plants together with eradication costs, Lantana mentioned in Eastern Transvaal: area infested with Bugweed, inkberry, brambles and Lantana 10823 ha; area cleared of same 2617 ha, cost 20.873 R |
| 1974/5 | Table 3.5 Eastern Transvaal Solanumspp, bramble, inkberry, wattle, Lantana, Mauritius thorn and Cetaria grass infest 15755 ha, cleared area 4342 ha, cost 33404 R (out of total annual expenditure on eradication of 209973 R). Northern Transvaal Solanumspp, wattle, Lantana, Mauritius thorn infest 1674 ha, cleared area 461 ha, cost 8096 R |
| 1975/6 | Table 3.5: Eastern Transvaal, Solanumspp, bramble, inkberry, wattle, Lantana, Mauritius thorn and Cetaria grass infest 19048 ha, cleared area 6371 ha, cost 62194 R. Northern Transvaal Solanumspp, wattle, Lantana, Mauritius thorn infest 2090 ha, cleared area 661 ha, cost 12674 R |
| 1976/7 | Table 3.5: Eastern Transvaal Solanumspp, bramble, inkberry, wattle, Lantana, Mauritius thorn and Setaria grass infest 16208 ha, cleared area 5773 ha, cost 79529 R. Northern Transvaal Solanumspp, wattle, Lantana, Mauritius thorn infest 2614 ha, cleared area 594 ha, cost 13927 R |
| 1977 | ‘While uncommon at present... *Lantana camara* represents a serious potential danger as it is difficult to control, being widely spread by birds. No effort should be spared to eradicate these undesirable plants.’ (study of Westfalia Estate on the North-Eastern Transvaal Escarpment) (Scheepers, 1977) |
| 1977 | No quantitative data on the extent or spread of Lantana in South Africa (Cilliers, 1977) |
| 1977/8 | ‘The control of Mauritius thorn, Bugweed and Lantana was still a problem in the Northern Trasnvaal Forest Region’ NB also controlling invader vegetation in conservation areas, but Lantana not mentioned. Western Cape Tsitsikamma Plantation Lantana, wattle, Eucalyptus, brambles 483 ha, 258 ha, 3139 R. Eastern Cape Oos-Kaap plantation Solanum, Cliffortia, wattle, Lantana 4360 ha, 1402 ha, 51183 R. Northern Transvaal Solanum, Mauritius thorn, Lantana, wattle 4434 ha, 935 ha 17978 R. |
| 1978 | Lantana listed as aggressive weed in forestry plantations with management guidelines (not nec. being fully controlled, 15% of the total labour costs) (see Marsh, 1978) |
| 1978-79 | Northern Transvaal Forest Region Mauritius thorn, antana (*Lantana camara*) and bugweed spread further despite considerable expenditure on the eradication of these species.’ Tsitsikamma, Acacia, Rubus, Eucapyptus, Hakea, Lantana 3917 ha, 2196 ha cleared, cost 24854 R. Eastern Transvaal, Setaria, Solanum, Phytolacca, Lantana, Caesalpinea, Acacia, 15854 ha, 7420 ha cleared, cost 119300 R. Eastern Cape conservation areas, Acacia, Lantana, Hakea, Opuntia occur in isolated groups, cost 3500 R to clear |
| 1979-1980 | Tsitsikamma, Acacia, Rubus, Eucapyptus, Hakea, Lantana 4829 ha, 3058 ha cleared, cost 140558 R. Eastern Transvaal, Setaria, Solanum, Phytolacca, Lantana, Caesalpinea, Acacia, 13423 ha, 5727 ha cleared, cost 76646 R. Eastern Cape conservation areas, Acacia, Lantana, Hakea, Opuntia occur in area covered unknown, 200 ha cleared, cost 9799 R to clear. |
| 1980/1 | ‘The invader vegetation problem is still increasing in the Eastern Transvaal Region. It is being controlled to a certain extent, but there is no possibility of eradication owing to the high cost and the extent of infestation. The problem with various invader plants in the Northern Transcaal Forest Region is still serious. Good progress was, however, made with the Lantana camara project and 106,9 ha was cleared in the Entabeni State Forest.’ Tsitsikamma, Acacia, Rubus, Eucapyptus, Hakea, Lantana 2955.6 ha, 569,4 ha cleared, cost 23144 R. Eastern Transvaal, Setaria, Solanum, Phytolacca, Lantana, Caesalpinea, Acacia, 14383 ha, 6542 ha cleared, cost 128108 R. Eastern Cape conservation areas, Acacia, Lantana, Hakea, Opuntia 1000 ha, 320 ha cleared, cost 10223 R to clear. |
| 1980 | Invader vegetation: *Lantana camara* listed (together with Rubus, so, Phytolaccadiandra and Solanummauritanum) as cause for increasing concern to tree growers – heavy cost of eradication. Methods inc herbicides, mowing, hoeing out, drying and burning – seldom removed in one operation (Department of Forestry, 1980). |
| 1982 | Several different cultivars of Lantana are invasive in SA (Spies and Stirton, 1982 a-c) |
| 1983 | Conservation of Agricultural Resources Act 1983, *Lantana camera* declared weed (Category 1 invasive species) in South Africa - not condoned on land within urban areas, must be eradicated or effectively controlled on farm units (control subject to The Conservation of Agricultural Resources Act – Act No 43 1983). Lantana, subject of herbicide registration and biocontrol investigation (University of Pretoria, 2011). |
| 1983 | Lantana has invaded about 2.2 million hectares of forest plantations, watercourses and savanna; mechanical and chemical control little effect (Cilliers, 1983) |
| 1986 | *Lantana camara* listed in ‘Problem plants of southern Africa’ (Wells et al., 1986). Undesirable characteristics: competitive (space, light, water, nutrients), may inhibit growth, replaces preferred vegetation (indigenous, grass), transforming (habitat, landscape), poisonous, thorny, obstructive (access, cultivation, vision), significantly increases erosion and decreases soil binding. |
| 1989 | Lantana camara 6th most common invasive species in roadside and veld habitats survey. Grows best in hot and humid coastal belt where it can form dense thickets, though has a wider climatic tolerance. Abundant along the coast south of Lake St Lucia but scarce north of this point. Abundant along the Pongola, Usutu and Ingwavuma Gorges after floods caused by cyclone Demonia in 1984 (MC Ward pers comm.) (Henderson, 1989) |
| 1995 | Lantana occurs length of southern coast and up into Mapumalanga, Guateng, Limpopo. Invades forest and plantation margins, savanna, water courses, roadsides and degraded land (Henderson, 1995) |
| 2000 | Research in Zululand, KZN and Mpumalanga; leaving woody vegetation (inc Lantana) on plantation plots led to greatest reduction in Pine growth. Lists *Solanum mauritianum*, *Lantana camara*, *Acacia mearnsii* and *Phytolacca octandra* as predominant woody plants encountered. Last 3 have extremely high growth and reproductive rates, rapidly occupy disturbed sites (Bromilow, 1997). Main mechanism of suppression – light availability. (Rolando & Little, 2000). |
| 2003 | Lantana identified as highest priority weed in South Africa (Robertson et al., 2003). |
| 2004 | *Lantana camara* forms closed canopy stands in Eastern Cape grasslands (Richardson & van Wilgen, 2004). |
| 2004 | Water is a critical resource in this semiarid region, and multiple invasive species, including *Lantana camara*, have substantially decreased available surface water and stream flow through their high evapotranspiration rates (Gorgens& van Wilgen, 2004). |

**References**

Baars JR, Neser S. 1999. Plant pathogens and biological control of weeds in South Africa: a review of projects and progress during the last decade. In: (eds. Olckers T & Hill MP) AfricanEntomology. Biological Control of Weeds in South Africa (1990-1998). African Entomology Memoir No. 1 Entomological Society of Southern Africa129-137.

Cilliers CJ. 1977. On the biological control of Lantana camara in South Africa. In: Proceedings of the second National Weeds Conference of South Africa. Stellenbosch, South Africa: Balkema, Cape Town, 341-344.

Cilliers CJ. 1983. The weed, Lantana camara L., and the insect natural enemies imported for its biological control into South Africa. Journal of the Entomological Society of Southern Africa 46: 131-138.

Codd LEW. 1951. Trees and shrubs of the Kruger National Park. In: Memoirs of the Botanical Survey of South Africa No. 26. Pretoria: Department of Agriculture, Division of Botany and Plant Pathology.

Cronk QCB, Fuller JL. 1995. Plant invaders. London: Chapman and Hall.

Day MD, Wiley CJ, Playford J, Zalucki MP. 2003. Lantana: Current Management, Status and Future Prospects. In: ACIAR Monograph 102. Canberra: Australian Centre for International Agricultural Research.

Department of Environmental Affairs SA. 2011. State of the environment: invasice species. In: http://soer.deat.gov.za/420.html.

Department of Forestry. 1980. Forestry in South Africa. Pretoria: Green Heritage Committee of the Forestry Council.

Department of Forestry.various. Forestry and Environmental Conservation Branch Annual Report. In.

Edwards D. 1967. A plant ecologyical survey of the Tugela river basin. In: Memoirs of the Botanical Survey of South Africa No. 36. Pretoria: Town and Regional Planning Commission, Natal.

Gorgens AHM, van Wilgen BW. 2004. Invasive alien plants and water resources in South Afrca: current understanding, predictive ability and research challenges. South African Journal of Science 100: 1381-1388.

Henderson L. 1989.Invasive alien woody plants of Natal and the north-eastern Orange Free State.Bothalia 19: 237-261.

Henderson L. 1995.Plant invaders of Southern Africa. A pocket field guide to the identification of 161 of the most important and potentially important alien species. Pretoria: Plant Protection Research Institute, Agricultural Research Council.

Henderson M, Anderson JG. 1966. Common weeds in South Africa. In: Memoirs of the Botanical Survey of South Africa No. 37. Pretoria: Department of Agricultural and Technical Services, Botanical Research Institute.

Kirby D, Shackleton SE, Gambiza J. In press. Invasive plants - friends or foes: Contribution of prickly pear (Opuntiaficus-indica) to livelihoods in Makana Municipality, Eastern Cape, South Africa. Development Southern Africa

Louw PGJ. 1950. Lantana as a weed. Farming in South Africa 25: 163-164.

Marr RS. 1964. Get rid of Lantana. Fmg. S. Afr.: 42-45.

Marsh EK. 1978. The cultivation and management of commercial pine plantations in South Africa. In: Forestry and Environmental Conservation Branch Bulletin 56. Pretoria: Department of Forestry.

McGibbon J. 1858. Catalogue of plants in the Botanic Garden, Cape Town. Cape Town: Salel Solomon.

Naude CP, Serfontein J. 1956. Eradication of Lantana and thorn trees.Farming in South Africa 31: 209-210.

Parsons WT, Cuthbertson EG. 2001. Noxious weeds of Australia. 2nd edn. Collingwood, Melbourne: CSIRO Publishing.

Pass MA, Heath T. 1977. On the biological control of Lantana camara in South Africa. In: Proceedings of the Second National Weeds Conference of South Africa. Stellenbosch 341-344.

Richardson D, van Wilgen BW. 2004. Invasive alien plants in South Africa: how well do we understand the ecological impacts? South African Journal of Science 100: 45-52.

Ritchie AH. 1943. Lantanin, the active principle of Lantana camara L. Part 1.-Isolation and preliminary results on the determination of its constitution. Onderstepoort Journal of Veterinary Science 18: 197-202.

Robertson MP, Villet MH, Fairbanks DHK, Henderson L, Higgins SI, Hoffmann JH, Le Maitre DC, Palmer AR, Riggs I, Shackleton CM, Zimmermann HG. 2003. A proposed prioritization system for the management of invasive alien plants in South Africa. South African Journal of Science 99: 37-43.

Rolando CA, Little KM. 2000. The impact of vegetation control on the establishment of pine at four sites in the summer rainfall region of South Africa: Institute for Commercial Forestry Research.

Samways MJ, Caldwell PM, Osborn R. 1996.Ground-living invertebrate assemblages in native, planted and invasive vegetation in South Africa. Agriculture Ecosystems & Environment 59: 19-32.

Scheepers JC. 1977. The vegetation of Westfalia Estate on the North-Eastern Transvaal Escarpment. In: Memoirs of the Botanical Survey of South Africa No. 42 (ed. Killick DJB). Pretoria: Department of Agriculture, Botanical Research Institute.

Stirton CH. 1977. Some thoughts on the polyploid Lantana camara L, (Verbenaccae). In: Proceedings of the Second National Weeds Conference. Stellenbosch, South Africa: Balkema, Cape Town, 321-340.

Thakur, M.L., M. Ahmad & R.K. Thakur. 1992. Lantana weed (Lantana camara var. aculeata Linn.) and its possible management through natural insect pests in India. Indian Forester 118: 466-488.

University of Pretoria. 2011. Projects of the Veterinary Science Library: Poisonous Plants - Lantana spp. http://www.ais.up.ac.za/vet/poison/bnames.htm#Lantana

Van Wilgen BW, Richardson DM, Le Maitre DC, Marais C, Magadlela D. 2001 The Economic Consequences of Alien Plant Invasions: Examples of Impacts and Approaches to Sustainable Management in South Africa. Environment, Development and Sustainability 3: 145-168.

Versfeld DB, Le Maitre DC, Chapman RA. 1998. Alien Invading Plants and Water Resources in South Africa: A preliminary assessment. Report TT99/98. In:Water Research Commission, Pretoria.

Wells MJ, Balsinhas AA, Joeff H, Engelberecht VM, Harding G, Stirton CH. 1986. A catelogue of problem plants in southern Africa. In: Memoirs of the Botanical Survey of South Africa. Pretoria: Botanical Research Institute, Department of Agriculture and Water Supply.

Wood JW. 1883. A guide to the Natal Botanical Gardens. Durban: Bennett & Davies.

Wood JW. 1910. Report on Natal Botanical Gardens and Colonial Herbarium for year 1909-1910. Durban: Bennett & Davies.
